# Sphingosine 1-phosphate receptor subtype 3 (S1P_3_) contributes to brain injury after transient focal cerebral ischemia via modulating microglial activation and their M1 polarization

**DOI:** 10.1186/s12974-018-1323-1

**Published:** 2018-10-10

**Authors:** Bhakta Prasad Gaire, Mi-Ryoung Song, Ji Woong Choi

**Affiliations:** 1 College of Pharmacy and Gachon Institute of Pharmaceutical Sciences, Gachon University, Incheon, 406-799 Republic of Korea; 2 School of Life Sciences, Gwangju Institute of Science and Technology, Buk-gu, Gwangju, 500-712 Republic of Korea

**Keywords:** Transient focal cerebral ischemia, S1P_3_, CAY10444, Microglial activation, M1 polarization, ERK1/2, p38 MAPK

## Abstract

**Background:**

The pathogenic roles of receptor-mediated sphingosine 1-phosphate (S1P) signaling in cerebral ischemia have been evidenced mainly through the efficacy of FTY720 that binds non-selectively to four of the five S1P receptors (S1P_1,3,4,5_). Recently, S1P_1_ and S1P_2_ were identified as specific receptor subtypes that contribute to brain injury in cerebral ischemia; however, the possible involvement of other S1P receptors remains unknown. S1P_3_ can be the candidate because of its upregulation in the ischemic brain, which was addressed in this study, along with underlying pathogenic mechanisms.

**Methods:**

We used transient middle cerebral artery occlusion/reperfusion (tMCAO), a mouse model of transient focal cerebral ischemia. To identify S1P_3_ as a pathogenic factor in cerebral ischemia, we employed a specific S1P_3_ antagonist, CAY10444. Brain damages were assessed by brain infarction, neurological score, and neurodegeneration. Histological assessment was carried out to determine microglial activation, morphological transformation, and proliferation. M1/M2 polarization and relevant signaling pathways were determined by biochemical and immunohistochemical analysis.

**Results:**

Inhibiting S1P_3_ immediately after reperfusion with CAY10444 significantly reduced tMCAO-induced brain infarction, neurological deficit, and neurodegeneration. When S1P_3_ activity was inhibited, the number of activated microglia was markedly decreased in both the periischemic and ischemic core regions in the ischemic brain 1 and 3 days following tMCAO. Moreover, inhibiting S1P_3_ significantly restored the microglial shape from amoeboid to ramified microglia in the ischemic core region 3 days after tMCAO, and it attenuated microglial proliferation in the ischemic brain. In addition to these changes, S1P_3_ signaling influenced the proinflammatory M1 polarization, but not M2. The S1P_3_-dependent regulation of M1 polarization was clearly shown in activated microglia, which was affirmed by determining the in vivo activation of microglial NF-κB signaling that is responsible for M1 and in vitro expression levels of proinflammatory cytokines in activated microglia. As downstream effector pathways in an ischemic brain, S1P_3_ influenced phosphorylation of ERK1/2, p38 MAPK, and Akt.

**Conclusions:**

This study identified S1P_3_ as a pathogenic mediator in an ischemic brain along with underlying mechanisms, involving its modulation of microglial activation and M1 polarization, further suggesting that S1P_3_ can be a therapeutic target for cerebral ischemia.

**Electronic supplementary material:**

The online version of this article (10.1186/s12974-018-1323-1) contains supplementary material, which is available to authorized users.

## Background

Sphingosine 1 phosphate (S1P), which is a bioactive sphingolipid, has been known to influence a variety of biological actions throughout the body [1]. These actions of S1P in various organs are mostly mediated by its five specific G-protein coupled receptors (S1P_1–5_) [1]. Based on the identified biological actions of S1P, a considerable effort has been made to develop a drug that targets S1P receptors, leading to the first successful output, FTY720 (fingolimod, Gilenya, Novartis), that binds non-selectively to 4 of the 5 S1P receptors after being phosphorylated [1] and is currently used for treatment of multiple sclerosis [2]. In addition to this success, FTY720 is now under clinical trials for the treatment of several disease types, including acute stroke, amyotrophic lateral sclerosis, schizophrenia, Rett syndrome, and glioblastoma [2], strongly suggesting that receptor-mediated S1P signaling can be a considerable drug target in different diseases. However, the S1P receptor subtypes involved in each disease type is still unclear. Even the efficacy of FTY720 has been assumed to be primarily mediated by S1P_1_, and no other subtypes targeted by FTY720 have been identified that mediate its efficacy.

Cerebral ischemia, which is caused by a sudden interruption of blood flow to the brain, is a disease type where S1P receptors become validated drug targets mainly due to the efficacy of FTY720. Numerous in vivo studies have been conducted to prove the neuroprotective effects of FTY720 in the brain against ischemic challenge [3–9]. FTY720 itself [10, 11] or combined with a thrombolytic agent [12] is under clinical trials for the treatment in acute stroke. Despite this validated efficacy, among the four S1P receptor subtypes targeted by FTY720, S1P_1_ is the only identified receptor subtype to be associated with cerebral ischemia [13], indicating the possible involvement of other subtypes of FTY720-relevant S1P receptors. Besides S1P_1_, S1P_2_ (which is not a target for FTY720) was also revealed to influence brain injury after ischemic challenge [14]. These two independent studies identified the importance of receptor-mediated S1P signaling in cerebral ischemia and further demonstrated the pathogenic roles of both receptor subtypes in this disease. Interestingly, the pathogenic roles of S1P_1_ in cerebral ischemia [13] demonstrated that FTY720’s efficacy in this disease is via its unique action as a functional antagonist for S1P_1_ [15, 16]. In addition to S1P_1_, FTY720-phosphate may also antagonize S1P_3_ because it reduced cellular responses through S1P-S1P_3_ signaling axis [17]. Furthermore, S1P_3_ was reported to be upregulated at mRNA levels in the brain after ischemic challenge [6]. This notion raised the possibility that S1P_3_ could be an additional pathogenic factor for cerebral ischemia, and FTY720’s efficacy in cerebral ischemia can also be mediated via suppressing S1P_3_. However, whether S1P_3_ influences brain injury in focal cerebral ischemia and the role of S1P_3_, pathogenic or neuroprotective, has not been identified.

In this study, we aimed to address the pathogenic role of S1P_3_ in transient focal cerebral ischemia with a mouse model of transient middle cerebral artery occlusion and reperfusion (tMCAO). To identify the role, we used a selective S1P_3_ antagonist, CAY10444, that was given to mice immediately after reperfusion. We then assessed brain damage such as brain infarction, neurological functional deficit, and neural cell death. We further assessed whether S1P_3_ influenced microglial activation and polarization, a core pathogenic event in cerebral ischemia, along with a clarification of S1P_3_-dependent effector pathways in the brain after tMCAO challenge.

## Methods

### Animals and surgical procedures

Male ICR mice (32 ± 2 g; 6 weeks old) were bought from the Orient Bio company (Korea) and housed under controlled environmental conditions of diurnal lighting (light on 07:00–19:00), temperature (22 ± 2 °C), and relative humidity (60 ± 10%). All animal handling and surgical procedures were carried out in accordance with the approved animal protocols specified by the Institutional Animal Care and Use Committee at Gachon University (Incheon, Republic of Korea) (no. of approved animal protocols: LCDI-2015–0048; LCDI-2014–0079). Following 1 week of laboratory acclimatization, the mice were challenged with tMCAO as described previously [18]. In brief, the mice were anesthetized with isoflurane (3% for induction and 1.5% for maintenance of anesthesia) in a N_2_O∶O_2_ (3∶1) mixture, and the right common carotid artery was isolated through a ventral neck incision. A silicone-coated 5–0 monofilament was introduced to the internal carotid artery from carotid bifurcation and advanced to occlude the middle cerebral artery (MCA). After 90 min of MCAO, the filament was withdrawn to allow complete reperfusion of the cerebral area. During surgery, rectal temperature was maintained at 37.0 ± 0.5 °C with a homoeothermic blanket. Sham-operated mice received similar surgical procedure except for the occlusion of MCA. After surgery, three mice were kept in a single cage; wet food and soft bedding were provided to minimize the suffering from the operation until they were sacrificed for brain sampling.

### CAY10444 administration

CAY10444 (Cayman chemical, MI, USA) was dissolved in 1:1 mixture of chremophore EL and 100% ethanol, diluted in water, and injected intraperitoneally to mice at 0.1, 0.2, and 0.5 mg/kg at the time of reperfusion. For the tMCAO group, equal volumes of the vehicle were injected.

### Neurological function assessment and brain infarction determination

Functional neurological deficit was assessed using modified neurological severity score (mNSS) scale to determine the motor, sensory, balance, and reflex disorder 24 h following MCAO, as described previously [18–20]. Following the neurological score assessment, the mice were sacrificed with CO_2_ exposure; their brains were quickly removed and sliced in the mice brain matrix at 2 mm thickness. The obtained brain slices were incubated with 2% 2,3,5-triphenyltetrazolium chloride (TTC) in physiological saline for 20 min at 37 °C. The TTC-stained brain slices were photographed, and the infarct area was calculated using ImageJ software (National Institute of Mental Health, Bethesda, MD).

### Histological analysis

#### Tissue preparation

Brain tissue samples for histological analysis were obtained 1 or 3 days after tMCAO. Mice were anesthetized with a mixture of Zoletil 50® (10 mg/kg, i.m.) and Rompun® (3 mg/kg, i.m.), and their brains were perfused with ice-cold phosphate-buffered saline (PBS; pH 7.4) followed by 4% paraformaldehyde. The brains were incubated in the same fixative solution overnight, cryoprotected with 30% sucrose, and cut into 20-μm sections using a microtome cryostat. To ensure anatomical similarity of brain regions, two coronal brain sections obtained from the rostral to middle portion of the striatum and the cortex of each mouse brain were used for histological evaluation. In a different set of experiments, the mice brains were transcardially washed with ice-cold PBS and the ipsilateral brain hemisphere was used for RNA and protein extraction.

#### Fluoro Jade B staining

In order to identify any degenerating neurons following the tMCAO challenge, Fluoro Jade B (FJB) histochemical staining was performed 1 day after tMCAO induction. Brain sections were sequentially immersed in ethanol series (100% for 3 min, and 70% and 30% for 1 min each), rinsed in deionized water, and oxidized in 0.06% *w*/*v* KMnO_4_. Then, sections were stained with 0.001% (*w*/*v*) FJB in 0.1% (*v*/*v*) acetic acid solution for 30 min, rinsed in deionized water, dried in a slide warmer, cleared in xylene, and then cover-slipped.

#### Iba1 or glial fibrillary acidic protein (GFAP) immunohistochemistry

To evaluate the effect of S1P_3_ activity on microglia or astrocyte activation, Iba1 or GFAP immunohistochemistry was performed 1 or 3 days after tMCAO. Brain sections were oxidized with 1% H_2_O_2_ in PBS for 15 min and blocked with 1% fetal bovine serum (FBS) in 0.3% Triton-X100 in PBS for 1 h to block non-specific protein binding. Then, the brain sections were incubated with primary antibody against Iba1 (1:500, Wako) or GFAP (1: 500, Invitrogen) overnight at 4 °C followed by anti-rabbit secondary antibody (1:200). Sections were exposed to avidin and biotinylated horse-radish peroxidase macromolecular complex (ABC) kit (1:100, Vector Labs) and visualized with 3, 3′-diaminobenzidine tetrahydrochloride (DAB) exposure (0.02% DAB and 0.01% H_2_O_2_ in 0.05 M TRIS solution), dehydrated with ethanol, cleared in xylene, and mounted using mounting media.

#### Iba1/NF-κB double-immunohistochemistry

In order to determine whether the NF-κB pathway is triggered in activated microglia after the tMCAO challenge, cryostat brain sections were processed for double immunolabeling using antibodies against NF-κB (p65) and Iba1. The sections were incubated with TRIS-EDTA solution at 100 °C for 30 min for antigen retrieval, blocked with 1% FBS in 0.3% Triton X-100, and labeled with rabbit NF-κB (p65) (1:100) antibody overnight at 4 °C. The sections were labeled with a biotinylated secondary antibody (1:200) followed by incubation with an ABC kit. The signals were visualized with DAB staining (0.02% DAB and 0.01% H_2_O_2_ for 2 min). The stained sections were then washed with PBS (3 × 5 min), blocked, and incubated with primary antibodies against Iba1 (1:500) overnight at 4 °C. Sections were then labeled with appropriate secondary antibodies conjugated with Cy3 (1:1000) and mounted with VECTA SHIELD mounting medium.

#### Bromodeoxyuridine (BrdU)/Iba1 immunofluorescence

The role of S1P_3_ activity on tMCAO-induced microglia proliferation was determined using Iba1/BrdU double immunofluorescence. BrdU (50 mg/kg in PBS, i.p.) was administered twice a day at 12-h intervals on the second and third day after tMCAO challenge. Brain sections were prepared for Iba1/BrdU immunofluorescence as described previously [18, 21].

#### Image preparation and quantification

The brain sections after staining or immunolabeling were photographed using bright-field and fluorescence microscopy (BX53T, Olympus, Japan) equipped with a DP72 camera. Representative images were prepared using Adobe Photoshop CS3. For quantification, three photographs were taken from different area of each region and the number of immunopositive cells was counted. Then, the average number of immunopositive cells from each region was expressed in per unit area (mm^2^).

### Western blot analysis

Ipsilateral brain hemispheres were obtained 24 h following tMCAO induction and triturated with neuronal protein extraction reagent (NPER); the obtained proteins was thus separated in a 10% SDS-PAGE system and transferred to the polyvinylidene difluoride membrane. The membrane was blocked with 5% skim milk to avoid non-specific protein bindings and incubated with primary antibodies against rabbit pAkt, Akt, pERK1/2, ERK1/2, pp38, p38 (Cell signaling, all at 1:1000 dilution), and mouse β-actin (Sigma Aldrich, 1:5000) overnight at 4 °C followed by incubation with respective secondary antibodies (Jackson ImmunoResearch, 1:10000) for 2 h at room temperature and visualized with enhanced chemiluminescence (ECL) solution. The band intensity of each protein was analyzed using ImageQuant (TM) TL software, normalized with β-actin, and then expressed as fold changes of the sham-operated group.

### Mouse primary microglia culture, CAY10444 treatment, and transfection with S1P_3_ shRNA

Primary microglial cells were obtained from the brain cortices of 1–2-day-old mouse pups as described previously [13]. The microglial cells were seeded on 6-well plates at a density of 1 × 10^5^ cells/well. Microglial cells were starved overnight and treated with CAY10444 (1 μM) or vehicle (0.1% DMSO in DMEM). Thirty minutes later, microglia were stimulated with lipopolysaccharides (LPS) (100 ng/ml) for additional 24 h. Alternatively, the shRNA targeted with S1P_3_ (shS1P_3_) receptor or non-targeted control shRNA was transfected into the cells in serum and antibiotic-free medium. After 6 h of incubation, the media were replaced with serum and antibiotic containing media for an additional 42 h. S1P_3_-infected microglial cells were then challenged with serum starvation for 12 h and stimulated with LPS, and then harvested for qRT-PCR analysis.

### Quantitative real-time polymerase chain reaction (qRT-PCR)

Total RNA was extracted from the ipsilateral hemisphere of mice brain and cultured microglia cells using TRIzol Reagent (Invitrogen). One microgram of total RNA was reverse transcribed (RT) to synthesize cDNA. The gene expression levels of the different markers of M1- and M2-polarized microglia were determined using the StepOnePlus™ qRT-PCR system (Applied Biosystems) with the FG Power SYBR Green PCR master mix (Life Technologies) and primer sets (Additional file 1: Table S1). β-actin was used as the housekeeping gene.

### Statistical analysis

All statistical tests were performed using Graph Pad Prism 5 (Graph Pad Software Inc., La Jolla, CA, USA), and the data are presented as mean ± S.E.M. One-way ANOVA followed by the Newman-Keuls post hoc test was used to compare the data among the multiple experimental groups, while comparisons between the two groups were performed using the Student’s *t* test. *p* < 0.05 was set as statistically significant.

## Results

### Suppression of S1P_3_ activity attenuates brain infarction and neurological deficit in tMCAO-challenged mice

**Fig. 1:**
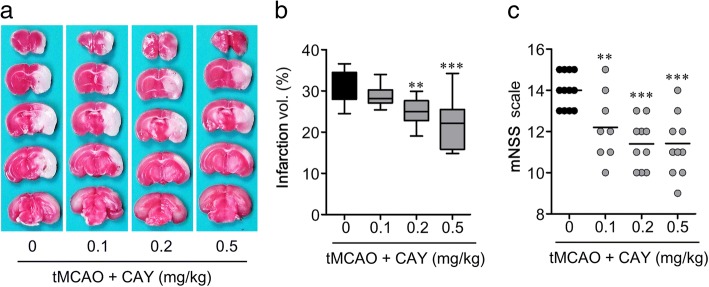
CAY10444 (CAY) administration attenuates tMCAO-induced brain infarction and neurological deficit. Mice were challenged with tMCAO, and CAY (0.1. 0.2, and 0.5 mg/kg) was administered intraperitoneally immediately after reperfusion. Brain damage was ascertained 24 h after tMCAO challenge (**a–c**). Effects of different dosage of CAY on infarct volume (**a, b**) and neurological function (**c**) were determined. Representative images of TTC-stained brain tissue (**a**), quantification of brain infarction (**b**), and neurological deficit (**c**) are shown. *n* = 10 ~ 12 mice per group. **p < 0.01 and ***p < 0.001 versus vehicle-administered tMCAO group

The vehicle-administered mice developed severe brain infarction in both the ischemic cortex and striatum 24 h after the tMCAO challenge (Fig. 1a, b). However, the mice administered with S1P_3_ antagonist (CAY10444) showed significantly decreased brain infarction in a dose-dependent manner (Fig. 1a, b). The brain infarction volume of the vehicle-administered tMCAO group was 31.20 ± 1.65%, whereas that in the different dosages of CAY10444-administered mice were 28.63 ± 0.97%, 25.20 ± 1.15%, and 21.96 ± 1.68% at 0.1, 0.2, and 0.5 mg/kg, respectively (Fig. 1a, b). The lowest dose of CAY10444 (0.1 mg/kg) was not effective, but 0.2 and 0.5 mg/kg were effective to attenuate the brain infarction. Similarly, the neurological deficit parameters reflecting motor, sensory, reflex, and balance disorders, as evidenced by mNSS analysis, were significantly improved in the CAY10444-administered mice compared to the vehicle-administered group (Fig. 1c). Among the tested doses, 0.5 mg/kg was found to be the most effective to attenuate brain infarction and neurological deficit; this dose was therefore chosen for the remaining experiments. The neuroprotective potential of CAY10444 in tMCAO-induced brain damage was further affirmed by analyzing the extent of neurodegeneration 24 h following ischemic challenge using FJB staining. CAY10444 reduced the extent of neuronal damage compared with vehicle exposure (Additional file 1: Figure S1). These results clearly demonstrated that the suppression of S1P_3_ activity attenuated tMCAO-induced brain damage, indicating the pathogenic role of S1P_3_ in cerebral ischemia.

### Suppression of S1P_3_ activity attenuates microglial activation and proliferation in the brain of tMCAO-challenged mouse

**Fig. 2:**
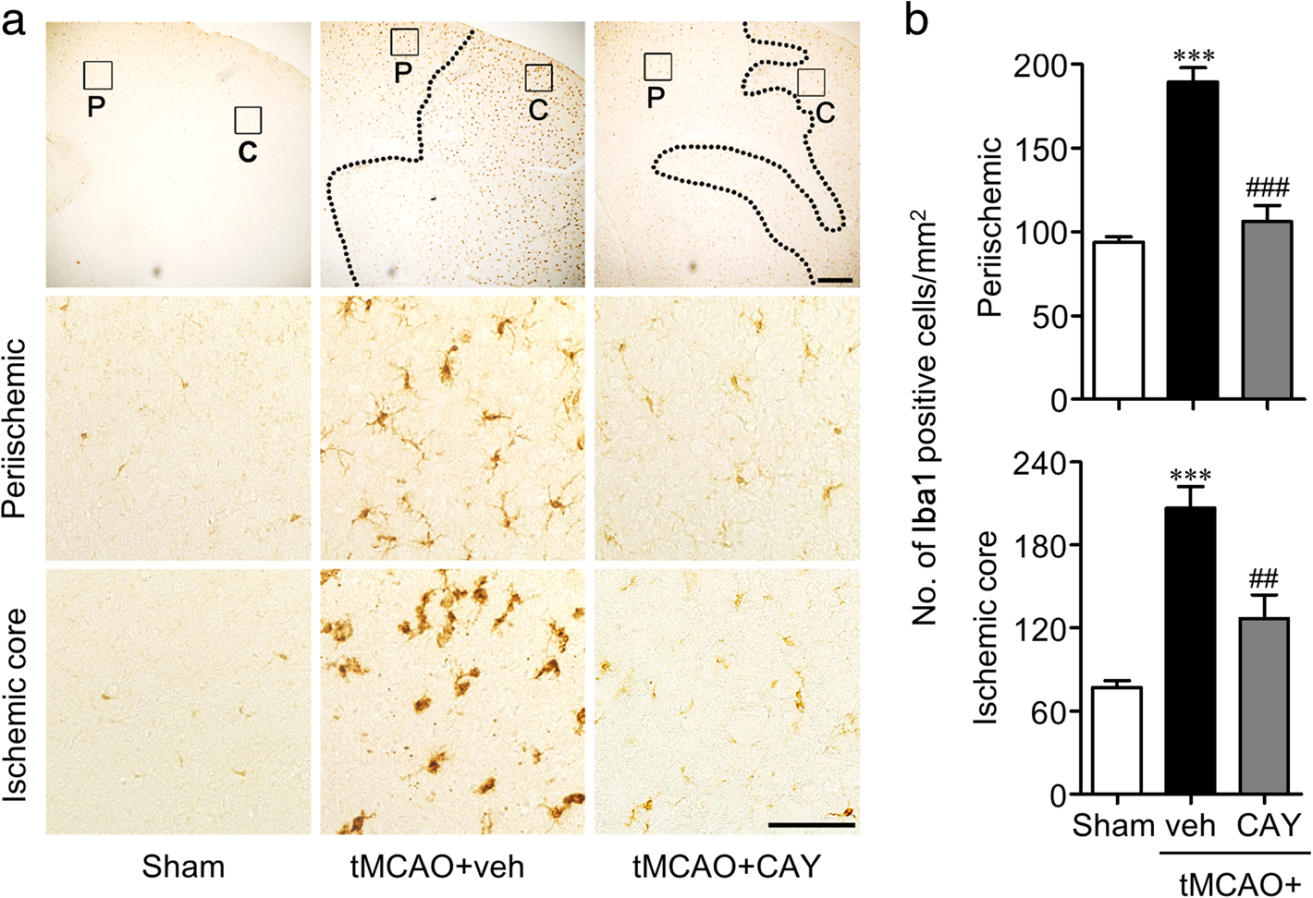
CAY10444 (CAY) administration attenuates tMCAO-induced microglia activation in 1 day post-ischemic brain. Mice were challenged with tMCAO, and CAY (0.5 mg/kg) was administered intraperitoneally immediately after reperfusion. The effect of CAY on microglial activation was determined by Iba1 immunohistochemistry in 1 day post-ischemic brain. **a** Representative images of Iba1-immunopositive cells in periischemic (P) and ischemic core (C) regions. Scale bars, 200 μm (top panels) and 50 μm (middle and bottom panels). **b** Quantification of the number of Iba1-immunopositive cells in both regions. *n* = 4 ~ 5 mice per group. ***p < 0.001 versus sham. ^##^*p* < 0.01 and ^###^*p* < 0.001 versus vehicle-administered tMCAO group (tMCAO+veh)

**Fig. 3:**
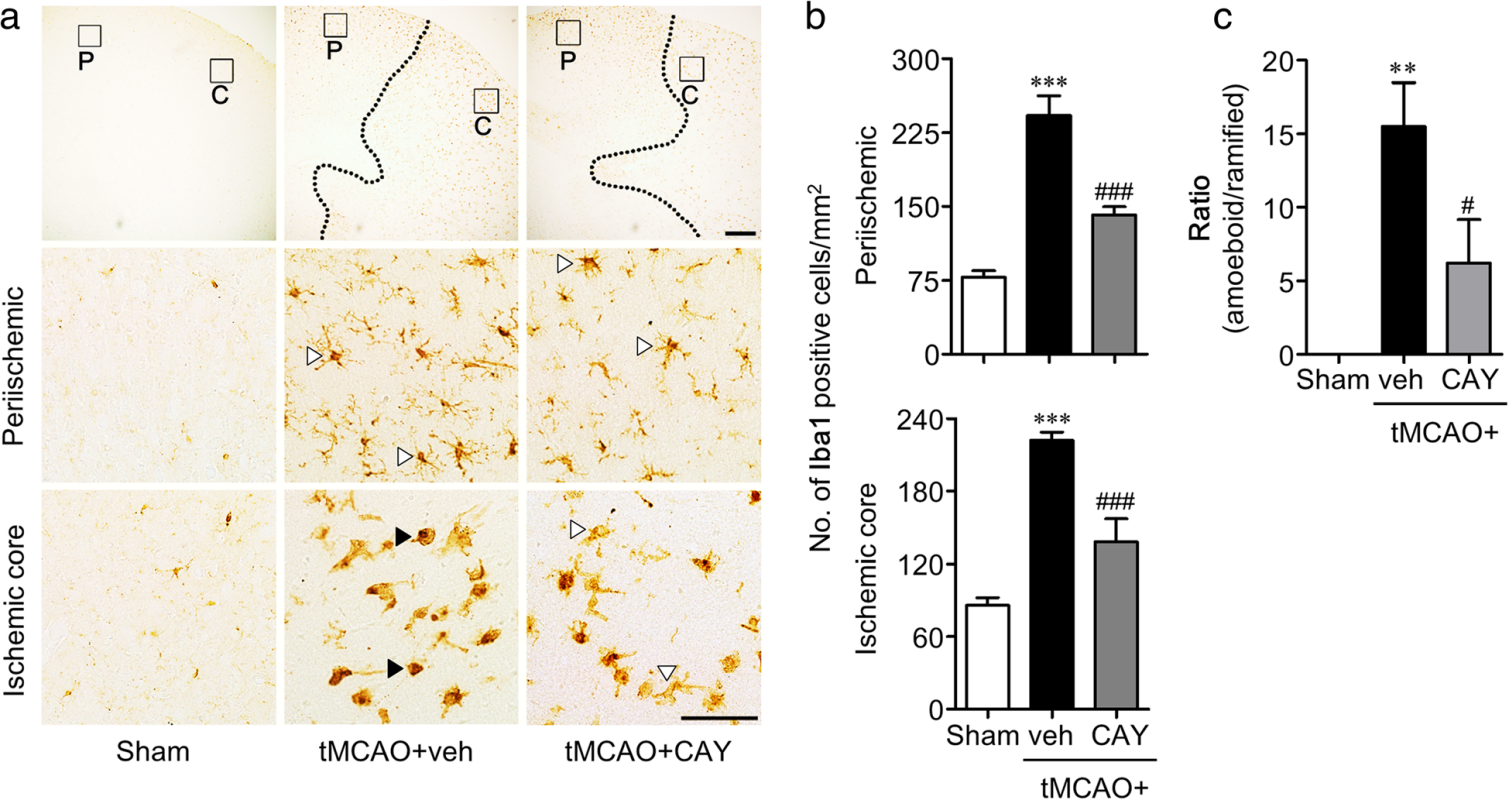
CAY10444 (CAY) administration attenuates tMCAO-induced microglia activation in 3 days post-ischemic brain. Mice were challenged with tMCAO, and CAY (0.5 mg/kg) was administered intraperitoneally immediately after reperfusion. The effect of CAY on microglial activation was determined by Iba1 immunohistochemistry in 3 days post-ischemic brain. **a** Representative images of Iba1-immunopositive cells in periischemic (P) and ischemic core (C) regions. Scale bars, 200 μm (top panels) and 50 μm (middle and bottom panels). **b** Quantification of the number of Iba1-immunopositive cells in both regions. **c** Quantification of morphological changes of Iba1-positive cells in ischemic core regions (ratio of amoeboid to ramified microglia). *n* = 5 mice per group. **p < 0.01 and ***p < 0.001 versus sham group. ^#^*p* < 0.05 and ^###^*p* < 0.001 versus vehicle-administered tMCAO group (tMCAO+veh)

Focal cerebral ischemia-induced microglial activation was analyzed in the brain through Iba1 immunohistochemistry 1 and 3 days following tMCAO challenge. The vehicle-administered tMCAO group showed the robust activation of microglia, as demonstrated by an increased number of Iba1-immunopositive cells in the ischemic hemisphere at both time points. CAY10444 administration significantly reduced the number of Iba1-immunopositive cells in a time- and region-dependent manner compared with the vehicle administration (Figs. 2 and 3). The number of activated microglia was significantly reduced in both the periischemic and ischemic core regions of the CAY10444-administered mice compared with the vehicle-administered mice at both time points (Figs. 2 and 3). Moreover, the number of amoeboid microglia in the ischemic core region was significantly reduced in the CAY10444-administered mice, as depicted by the reduced ratio of amoeboid/ramified microglia (Fig. 3c). These data demonstrated that suppressing S1P_3_ activity in an ischemic brain not only attenuated the activation of microglia, but also reduced the morphological transformation of ramified microglia to amoeboid microglia.

**Fig. 4:**
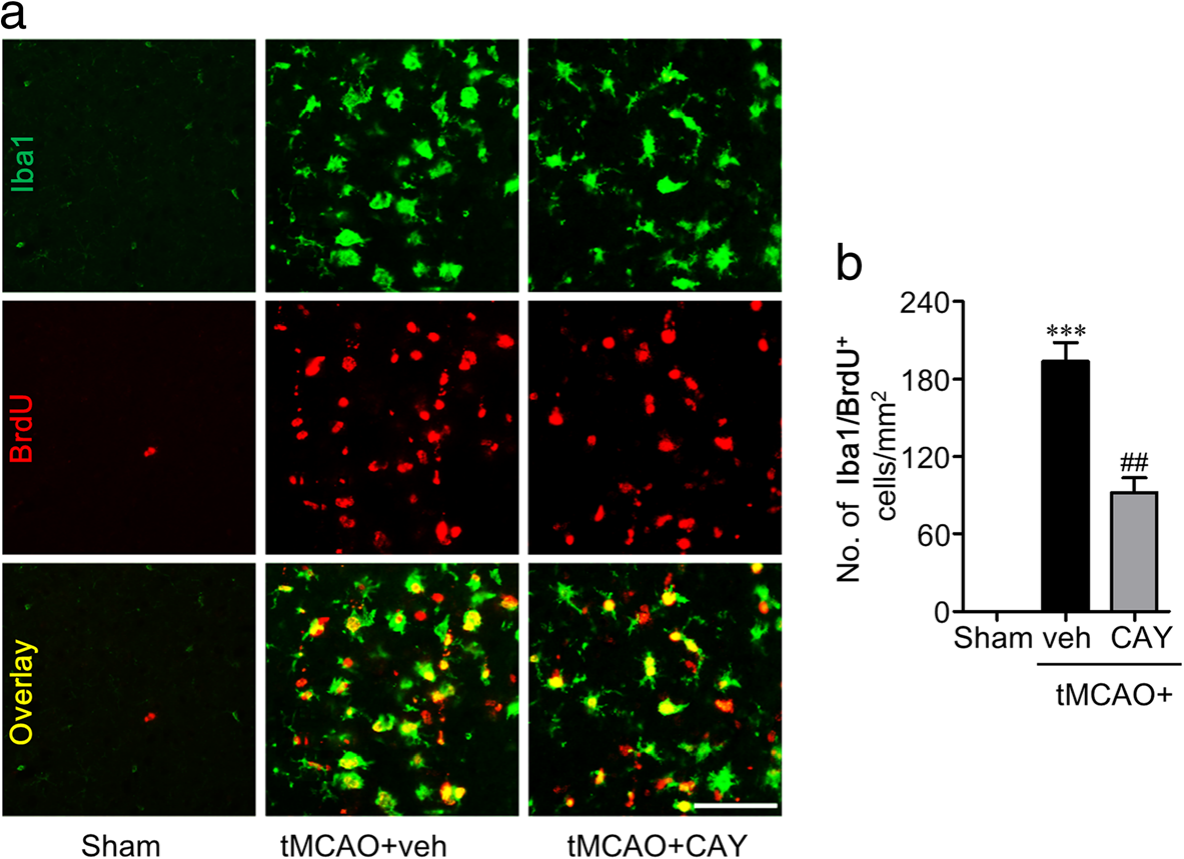
CAY10444 (CAY) administration attenuates tMCAO-induced microglia proliferation in 3 days post-ischemic brain. Mice were challenged with tMCAO, and CAY (0.5 mg/kg) was administered intraperitoneally immediately after reperfusion. The effect of CAY on microglial proliferation was determined by BrdU/Iba1 double immunofluorescence analysis in 3 days post-ischemic brain. **a** Representative images of BrdU/Iba1 double-immunopositive cells in marginal zone. Scale bars, 50 μm. **b** Quantification of the number of BrdU/Iba1-immunopositive cells. *n* = 5 mice per group. ***p < 0.001 versus sham group. ^##^*p* < 0.01 versus vehicle-administered tMCAO group (tMCAO+veh)

The brain resident microglia proliferated during the first week following the ischemic challenge, and these newly born microglia may participate in inflammatory responses [22]. To analyze the regulatory roles of S1P_3_ on microglial proliferation in the ischemic brain, we performed double immunofluorescence for BrdU and Iba1 in the brain 3 days after the tMCAO challenge. Microglial proliferation was obviously observed in the ischemic penumbra region of the vehicle-administered tMCAO group as evidenced by the increased number of BrdU/Iba1 double-immunopositive cells. The administration of CAY10444 significantly decreased the number of BrdU/Iba1 double-immunopositive cells compared with the vehicle administration (Fig. 4a, b), demonstrating that S1P_3_ is involved in microglial proliferation following ischemic challenge.

Besides microglial activation, astrogliosis is another core pathogenesis in cerebral ischemia [23], and S1P_3_ regulates inflammatory responses in activated astrocytes [24]. In this study, we also determined whether suppressing S1P_3_ activity reduced astrogliosis following ischemic challenge through GFAP immunohistochemistry. The vehicle-administered mice developed a significant astrogliosis in the corpus callosum as evidenced by the increased number of GFAP-immunopositive cells 1 and 3 days after the tMCAO challenge. CAY10444 administration significantly reduced the number of GFAP-immunopositive cells at both time points. In addition, the morphology of astrocytes was transformed towards reactive phenotype, particularly, 3 days after the tMCAO challenge, which was markedly attenuated by CAY10444 administration (Additional file 1: Figure S3). These results demonstrated that S1P_3_ signaling also regulated astrogliosis in the ischemic brain.

### S1P_3_ regulates microglial M1 polarization in the brain of tMCAO-challenged mouse

**Fig. 5:**
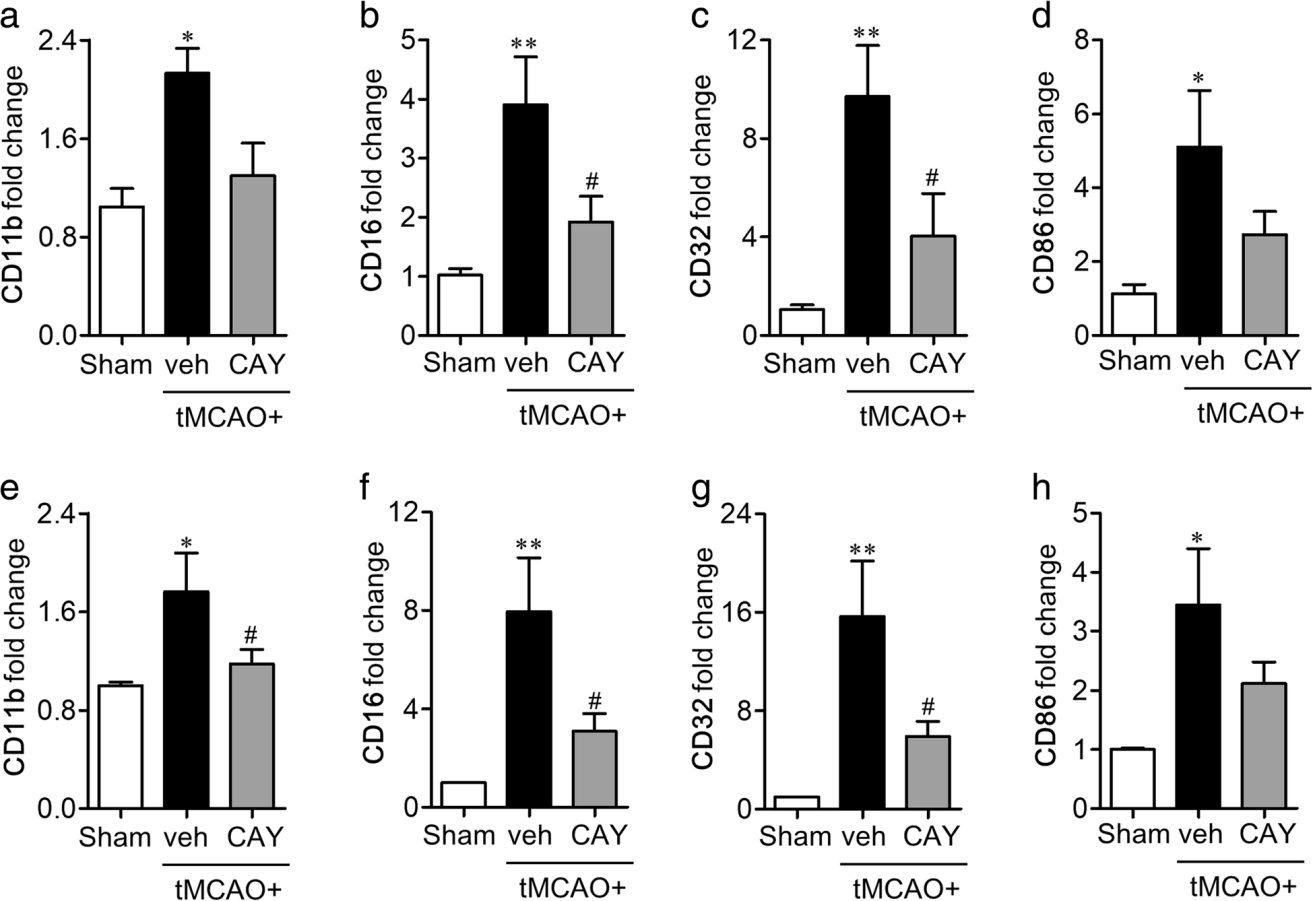
CAY10444 (CAY) administration attenuates expression level of surface markers of M1 polarization in post-ischemic brain. Mice were challenged with tMCAO, and CAY (0.5 mg/kg) was administered intraperitoneally immediately after reperfusion. The effect of CAY on mRNA expression of M1 surface markers in 1 day (**a–d**) and 3 days (**e–h**) post-ischemic brain was determined by qRT-PCR analysis. *n* = 5 mice per group. *p < 0.05 and **p < 0.01 versus sham group. ^#^*p* < 0.05 versus vehicle-administered tMCAO group (tMCAO+veh)

**Fig. 6:**
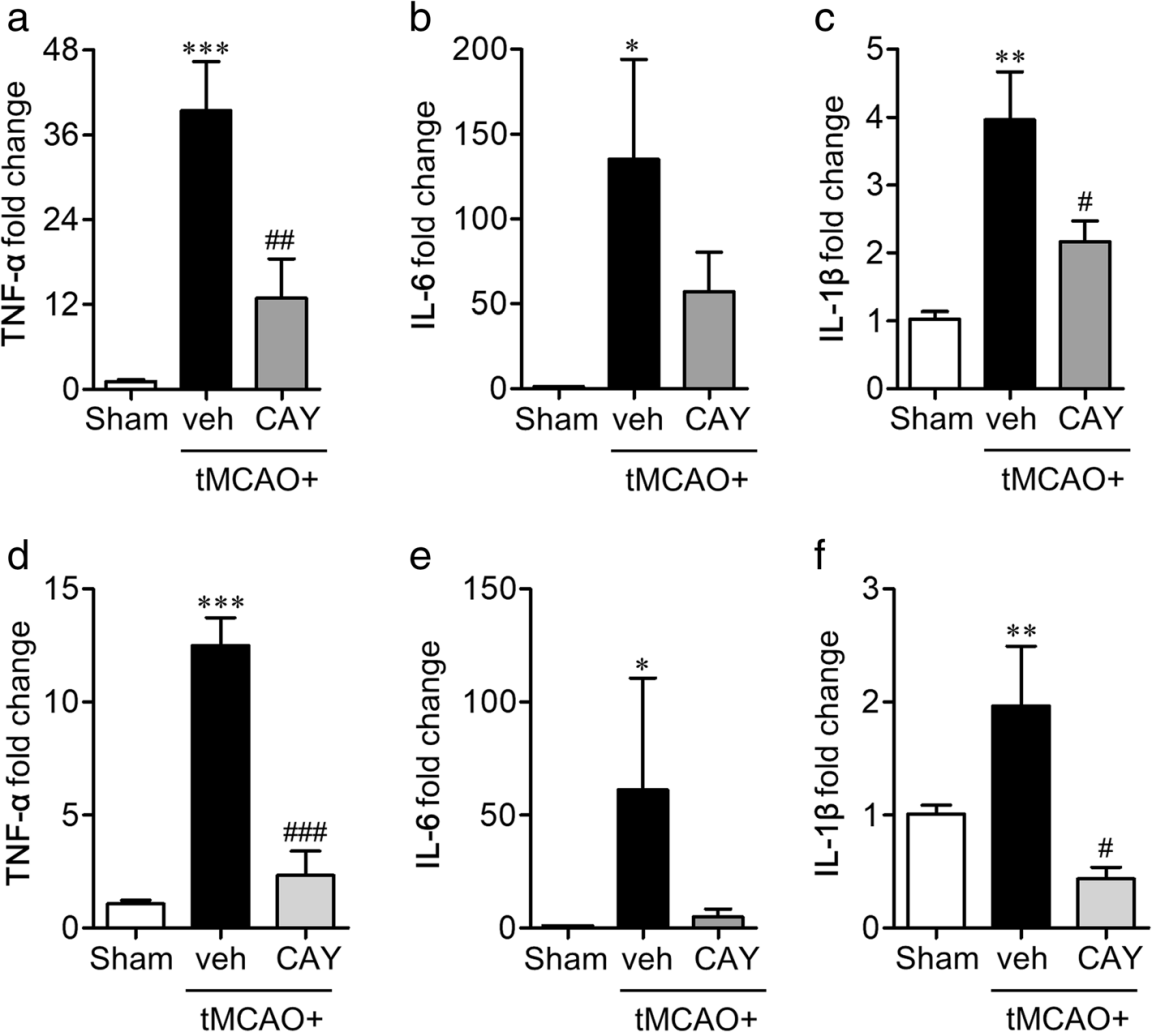
CAY10444 (CAY) administration attenuates expression level of soluble markers of M1 polarization in post-ischemic brain. Mice were challenged with tMCAO, and CAY (0.5 mg/kg) was administered intraperitoneally immediately after reperfusion. The effect of CAY on mRNA expression of M1 soluble markers in 1 day (**a–d**) and 3 days (**e–h**) post-ischemic brain was determined by qRT-PCR analysis. *n* = 5 mice per group. *p < 0.05, **p < 0.01, and ***p < 0.001 versus sham group. ^#^*p* < 0.05, ^##^*p* < 0.01, and ^###^*p* < 0.001versus vehicle-administered tMCAO group (tMCAO+veh)

Following ischemic injury, activated microglia become polarized into two distinct phenotypes, broadly known as proinflammatory M1 and anti-inflammatory M2 phenotypes [25]. In order to identify the association between S1P_3_ activity and M1/M2 polarization in the ischemic brain, the mRNA expression levels of different markers, both surface and soluble, of M1 and M2 polarization were determined. The mRNA expression levels of M1 surface markers (CD11b, CD16, CD32, and CD86) were significantly upregulated 1 and 3 days following the tMCAO challenge (Fig. 5). The upregulated surface markers of M1 polarization in the ischemic brain, such as CD16 and CD32, were significantly downregulated in the CAY10444-administered mice 1 day after the ischemic challenge (Fig. 5a–d). Similarly, CAY10444 administration significantly downregulated the mRNA expression levels of M1 surface markers (CD11b, CD16, and CD32) 3 days after the tMCAO challenge (Fig. 5e–h). We then determined whether S1P_3_ also regulated the expression of soluble markers that are functionally more important M1 markers. The administration of CAY10444 significantly reduced the mRNA expression levels of the proinflammatory cytokines, such as TNF-α and IL-1β, but not IL-6 (Fig. 6a–c) in the 1-day post-ischemic brain, which were reproduced in the 3-day post-ischemic brain (Fig. 6d–f). These data demonstrated that S1P_3_ triggered the proinflammatory responses of M1-polarized cells in the ischemic brain. We further determined whether S1P_3_, in the ischemic brain, had a role in the anti-inflammatory M2 polarization. However, the administration of CAY10444 did not alter the gene expression levels of the M2 markers (Arg1, CCL-22, CD206, TGF-β, and Ym-1) at both day 1 (Additional file 1: Figure S2a–e) and day 3 (Additional file 1: Figure S2f–j) following the ischemic challenge, suggesting that S1P_3_ in an ischemic brain is mainly associated with M1 polarization rather than M2 polarization.

**Fig. 7:**
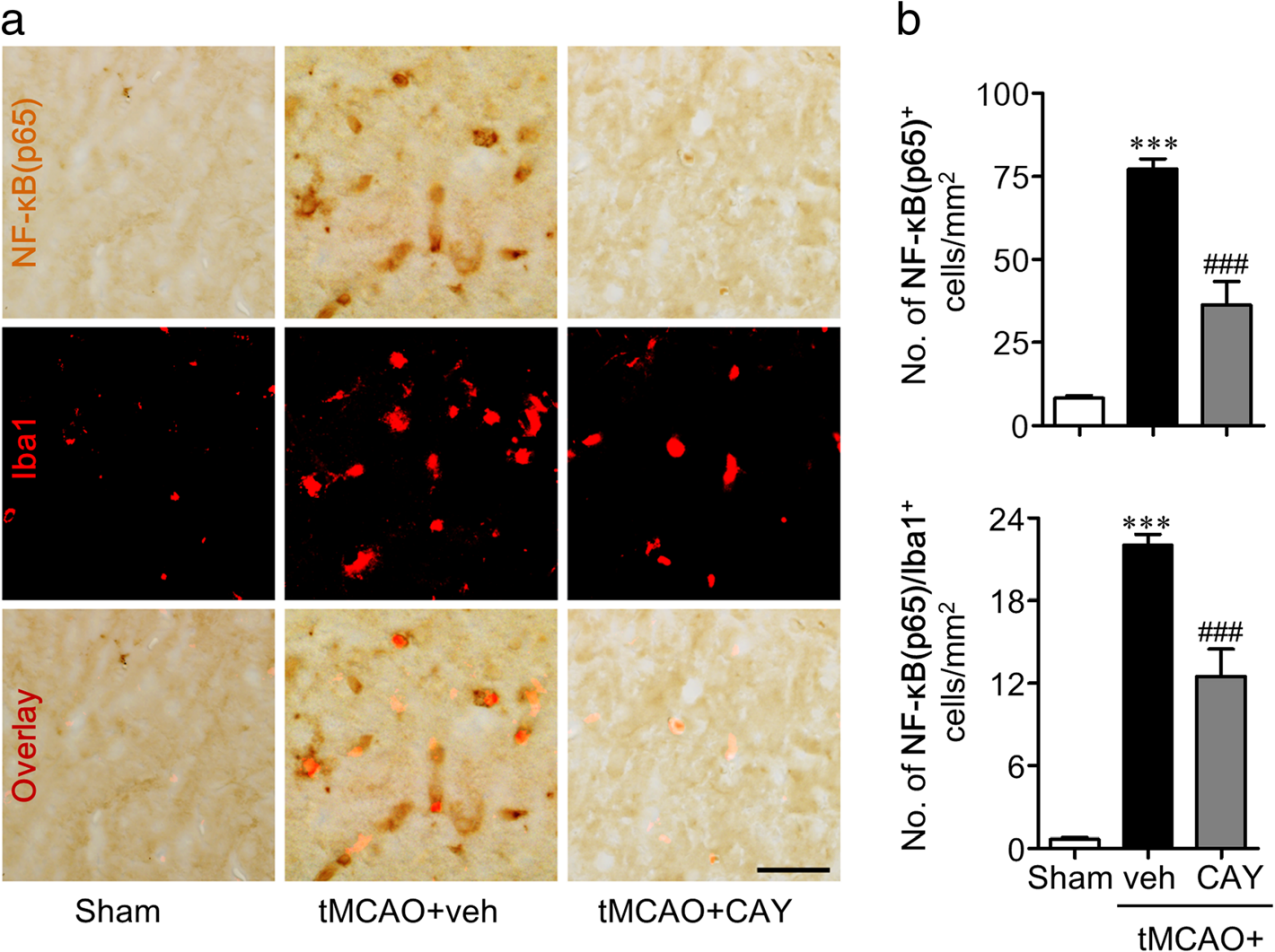
CAY10444 (CAY) administration attenuates tMCAO-induced microglial NF-κB expression in post-ischemic brain. Mice were challenged with tMCAO, and CAY (0.5 mg/kg) was administered intraperitoneally immediately after reperfusion. The effect of CAY on NF-κB expression in activated microglia was determined by NF-κB(p65)/Iba1 double immunohistochemical analysis in 1 day post-ischemic brain. **a** Representative images of NF-κB(p65)/Iba1-immunopositive cells in ischemic core regions. Scale bars, 50 μm. **b** Quantification of the number of NF-κB(p65)- and NF-κB(p65)/Iba1-immunopositive cells. *n* = 4~ 5 mice per group. ***p < 0.001 versus sham group and ^###^*p* < 0.001 versus vehicle-administered tMCAO group (tMCAO+veh)

**Fig. 8:**
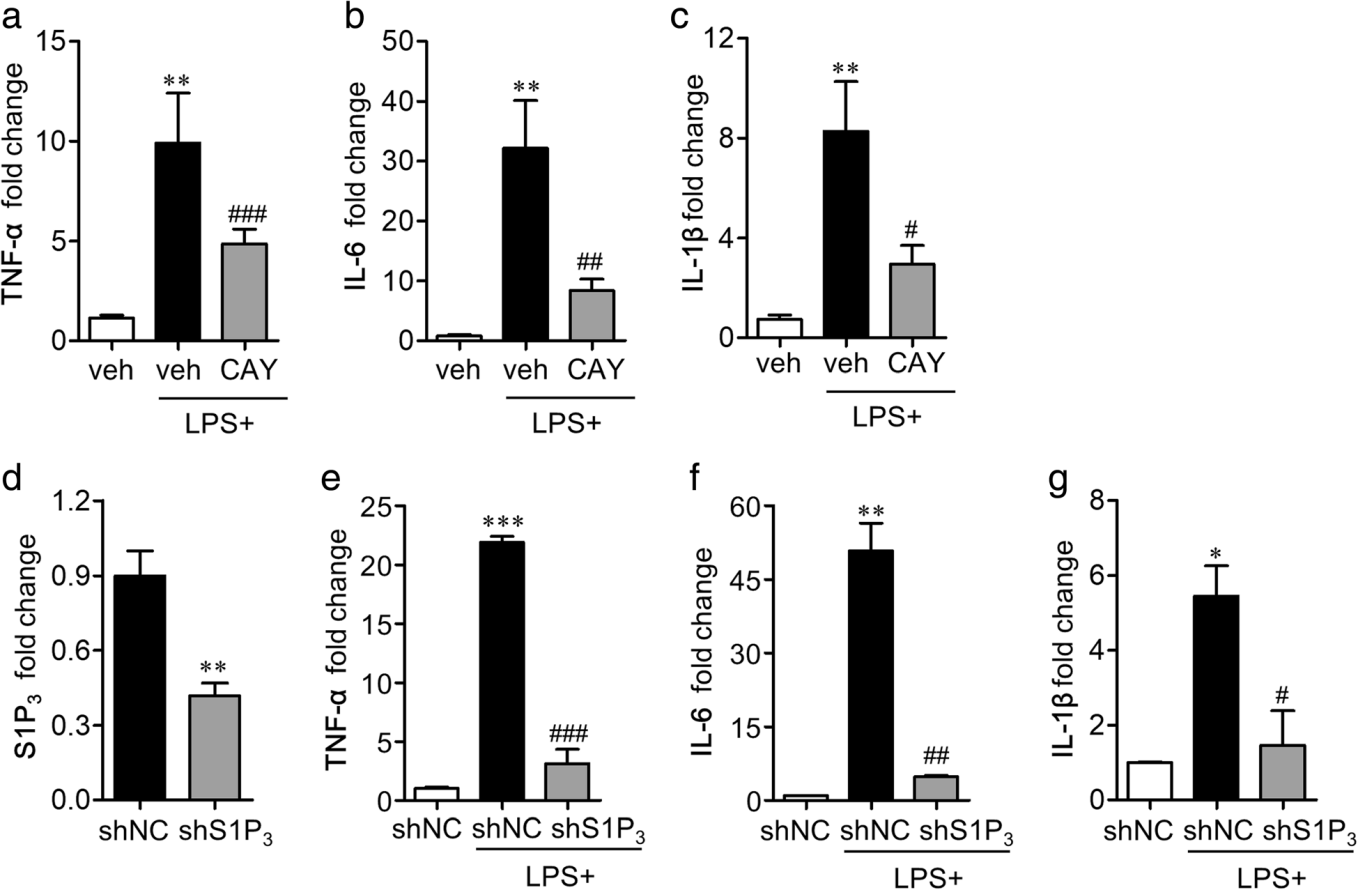
Suppression of S1P_3_ activity attenuates proinflammatory cytokine expression in LPS-stimulated mouse primary microglial cells. Mouse primary microglial cells were stimulated with LPS (100 ng/ml) 30 min after CAY (1 μM) treatment. Alternatively, cells were stimulated with LPS 2 days after transfection with S1P_3_ shRNA (shS1P_3_) or non-target control shRNA (shNC). **a–c** The mRNA expression levels of TNF-α, IL-6, and IL-1β were determined 24 h after LPS treatment using qRT-PCR analysis. *n* = 4 per group. **p < 0.01 versus vehicle-treated cells. ^#^*p* < 0.05, ^##^*p* < 0.01, and ^###^*p* < 0.001 versus LPS-stimulated cells. **d–g** Effects of S1P_3_ knockdown (**d**) on mRNA expression levels of TNF-α, IL-6, and IL-1β (**e–g**) were determined 24 h after LPS treatment in S1P_3_ shRNA and shNC transfected cells through qRT-PCR. *n* = 3 per group. **p < 0.01 versus shNC in d. *p < 0.05, **p < 0.01, and ***p < 0.001 versus vehicle-treated cells (**e**–**g**). ^#^*p* < 0.05, ^##^*p* < 0.01, and ^###^*p* < 0.001 versus LPS-stimulated cells

The M1 polarization is closely related to NF-κB signaling as the expression of most of the soluble M1 markers are dependent on a transcriptional activation of NF-κB. S1P_3_ was also found to regulate microglial activation and M1 polarization following ischemic injury in this study. Therefore, we tried to correlate the roles of S1P_3_ with NF-κB activation, especially in activated microglia, which was addressed by double immunolabeling for NF-κB(p65) and Iba1 1 day after the ischemic challenge. The vehicle-administered tMCAO group showed an enhanced expression of NF-κB(p65) which are easily identified in Iba1-immunopositive cells in the ischemic core region (Fig. 7a, b). CAY10444 administration significantly decreased the number of NF-κB(p65)-immunopositive cells or NF-κB(p65)/Iba1 double-immunopositive cells (Fig. 7a, b). These data further demonstrated that S1P_3_ in the ischemic brain mediated the M1 polarization through the activation of NF-κB signaling, in particular, in activated microglia. The regulatory role of S1P_3_ on M1 microglial polarization was reaffirmed using LPS-stimulated mouse primary microglia. For this purpose, we used LPS because LPS is a well-known stimulus to induce M1 polarization of microglia [26, 27]. The mRNA expression levels of M1-soluble markers (TNF-α, IL-6, and IL-1β) were significantly upregulated in LPS-treated cells. Suppressing S1P_3_ activity either pharmacologically, using CAY10444 (Fig. 8a–c), or genetically, using S1P_3_-specific shRNA lentivirus (Fig. 8d–g), attenuated the expression of these M1 markers. These data ensured that S1P_3_ in the ischemic brain might be associated with the inflammatory M1 polarization of activated microglia.

### S1P_3_ activity in ischemic brain was linked with activation of ERK1/2, p38 MAPK, and Akt effector pathways

**Fig. 9:**
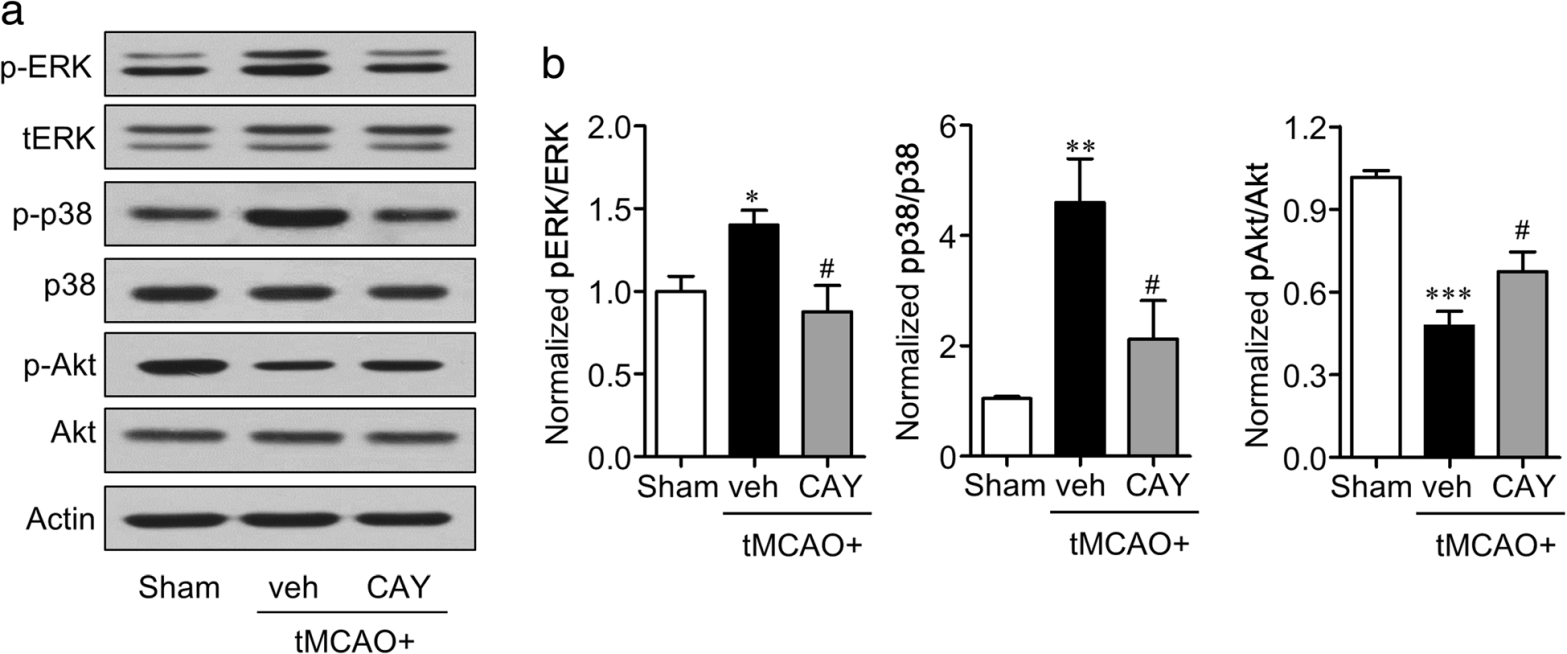
CAY10444 (CAY) administration alters tMCAO-induced Akt and MAPK expression in post-ischemic brain. Mice were challenged with tMCAO, and CAY (0.5 mg/kg) was administered intraperitoneally immediately after reperfusion. The effect of CAY on expression in MAPKs and Akt was determined by Western blot analysis. **a** Representative Western blots. **b** Quantification. *n* = 4 mice per group. *p < 0.05, **p < 0.01, and ***p < 0.001 versus sham group, and ^#^*p* < 0.05 vehicle-treated tMCAO (tMCAO+veh)

Microglial activation and their phenotype shift towards M1 polarization are linked to several signaling molecules, including ERK1/2, p38 MAPK, and PI3K/Akt [28–31]. Additionally, these signaling pathways function as G_i_ protein-associated effector systems under S1P_3_ activation [1]. Therefore, we determined whether S1P_3_ influenced the activation of these signaling components in an ischemic brain 24 h after tMCAO. In the ischemic brain, ERK1/2 and p38 MAPKs were significantly activated, as assessed by Western blotting for their phosphorylated forms (Fig. 9a, b). When S1P_3_ activity was blocked by CAY10444 administration, the increased phosphorylation of ERK1/2 and p38 MAPKs was significantly attenuated (Fig. 9a, b). Akt phosphorylation was reduced in the ischemic brain, and this reduction was significantly reversed by S1P_3_ antagonism (Fig. 9a, b), further implying the neurotoxic roles of S1P_3_ following the ischemic challenge because Akt phosphorylation is a well-known survival factor [32]. These data demonstrated that S1P_3_ influenced the activation of ERK1/2 and p38 MAPKs as well as the inactivation of Akt as downstream signaling cascades in cerebral ischemia.

## Discussion

In the current study, we identified S1P_3_ as another S1P receptor subtype that triggers pathogenesis in transient focal cerebral ischemia along with mechanistic features, particularly in terms of microglial biology, and the effector signaling pathways after S1P_3_ activation. Suppression of S1P_3_ activity after tMCAO by its specific antagonist results in attenuation of brain damages. The pathogenic roles of S1P_3_ in the ischemic brain are closely associated with microglial activation, involving an increased number of activated microglia, morphological transformation into amoeboid shape, and microglial proliferation. In addition, S1P_3_ regulates M1 microglial polarization, but not M2 polarization, in the ischemic brain because inhibiting S1P_3_ after tMCAO weakened the characteristics of M1 polarization without any influence on the M2 markers. These biological roles were further supported in vitro using LPS-stimulated primary microglia. Finally, PI3K/Akt, ERK1/2 MAPK, and p38 MAPK pathways were identified as effector pathways after S1P_3_ activation in the ischemic brain.

The use of receptor-mediated S1P signaling has been assumed as a possible therapeutic strategy to overcome cerebral ischemia because FTY720, which is a non-selective modulator of 4 of 5 S1P receptors after being phosphorylated, exerts neuroprotective effects in rodent models [3–8]. Currently, FTY720 is under clinical trial for the treatment of acute stroke [10, 11], and another trial for acute ischemic stroke is underway to determine its clinical efficacy in combination with a thrombolytic therapy, alteplase [12]. Despite these successful efforts, until recently, which S1P receptor subtypes are actual mediators for FTY720’s efficacy has remained uncertain. Our previous report proposed the first possibility for this, demonstrating S1P_1_ as a pathogenic factor in focal cerebral ischemia using a mouse model for transient focal cerebral ischemia [13]. The current study identified S1P_3_ as an additional S1P receptor subtype to mediate brain injury in cerebral ischemia. Notably, it has been discovered that FTY720-phosphate acts as a functional antagonist for S1P_1_ [15, 16] and possibly for S1P_3_. Even with no direct evidence for the latter, a few findings indicate that FTY720-phosphate antagonizes S1P_3_ signaling. Either FTY720-phosphate or TY-52156 (a selective S1P_3_ antagonist) reduced p-selectin production and leucocyte rolling via S1P-S1P_3_ signaling axis, which was reaffirmed in S1P_3_ knockouts [17]. FTY720-phosphate was also reported to antagonize G_q_-mediated signaling pathway under S1P_3_ activation [33]. Considering the inhibitory roles of FTY720-phosphate for S1P_1_ and S1P_3_, our previous and current in vivo findings strongly indicate that the reported FTY720’s efficacy in cerebral ischemia may be through suppressing at least the S1P_1_ and S1P_3_ activities. Besides FTY720-relevant target receptors, S1P_2_ was also identified to mediate brain injury in cerebral ischemia through the disruption of vascular integrity in the ischemic brain [14], even though it is not a target for FTY720-phosphate. Therefore, three subtypes of S1P receptors have been identified as pathogenic factors for cerebral ischemia. However, it is still unclear whether the mediation of the brain injury in the cerebral ischemia differs among the receptor subtypes and whether additional S1P receptor subtypes participate, such as S1P_4_ or S1P_5_.

Despite the clear pathogenic role of S1P_3_ in the brain, its roles in ischemic conditions seem to be tissue-specific. In fact, earlier studies reported controversial roles of S1P_3_ in non-neural ischemic models: protective or harmful. In the heart, the deletion of both S1P_2_ and S1P_3_ was shown to aggravate myocardial infarction in mice, which supported the cardioprotective role of S1P_3_ [34]. In the kidneys, however, S1P_3_ was shown to be associated with tissue injury after ischemic challenge. Deletion of bone marrow S1P_3_ attenuated tissue damage following renal ischemia/reperfusion, in which its deletion reduced the expression levels of proinflammatory cytokines and increased the expression levels of anti-inflammatory cytokines [35, 36]. These disparate roles for S1P receptors were similarly observed in the case of S1P_1_. Renal injury after ischemic challenge was reduced or exacerbated by exposure to an S1P_1_ agonist [37] or endothelial S1P_1_ deletion [38]. However, in the brain, S1P_1_ knockdown reduced brain injury after ischemic challenge [13]. Regardless of the different roles of receptor-mediated S1P signaling in non-neural tissues, it should be noted that all three identified S1P receptors (S1P_1_, S1P_2_, and S1P_3_) mediate pathogenesis in ischemic brain.

In this study, we have used CAY10444 to address the role of S1P_3_ in cerebral ischemia because CAY10444 has been widely used as a specific antagonist for S1P_3_ [39–41]. But, additional possible modes of actions of CAY10444 were suggested, which included S1P_2_, P2 receptor, or α_1A_-adrenoceptor [42]. CAY10444 at 10 μM blocked the S1P_2_- and S1P_3_-mediated increase in the intracellular calcium levels in Chinese hamster ovary cells. This inhibitory effect of CAY10444 was also mediated through the stimulation of P2 receptor or α_1A_-adrenoceptor [42]. These findings indicate that CAY10444 could also act as an antagonist for S1P_2_ and an agonist for P2 receptor or α_1A_-adrenoceptor. The latter agonistic property could be excluded in the protective effects of CAY10444 against cerebral ischemia: the association of α_1A_-adrenoceptor with cerebral ischemia is unclear and suppressing P2 receptor is neuroprotective in this disease [43, 44]. Unlikely, S1P_2_ could mediate the neuroprotective effects of CAY10444 in cerebral ischemia because S1P_2_ was reported as a pathogenic factor in this disease [14]. However, it is also possible that CAY10444’s efficacy is solely mediated through S1P_3_ in cerebral ischemia. In renal ischemic injury, blocking S1P_2_ activity by JTE013 resulted in renoprotection, whereas CAY10444 did not [45]. The latter indicates that CAY10444 does not act as S1P_2_ antagonist. It would be tempting to address these opposite notions using genetic tools such as knockout mice for S1P_3_ in future studies.

The neuroharmful role of S1P_3_ in the ischemic brain appears to be associated with the activation of brain residence microglia, which is a common pathogenic event in several central nervous system (CNS) disorders, including stroke [46, 47]. Previously, receptor-mediated S1P signaling was reported to be involved in microglial activation through both in vitro and in vivo studies [6, 48]. Recently, we identified that S1P_1_-mediated brain damage after focal cerebral ischemia was mainly mediated through microglial activation [13]. In this study, we identified that S1P_3_ was also associated with microglial activation: inhibiting S1P_3_ using its specific antagonist reduced the number of activated microglia in the ischemic brain, in both a time- and region-dependent manner. Furthermore, the suppression of S1P_3_ activity in the ischemic brain attenuated microglial proliferation. In addition to the increase in the population, S1P_3_ in the ischemic brain was closely associated with the morphological transformation of activated microglia. In the ischemic core regions 3 days or more after ischemic challenge, most of the activated microglia were amoeboid shaped and were mainly responsible for neuronal damage in ischemic brain by releasing several proinflammatory mediators [49, 50]. We demonstrated that inhibiting S1P_3_ resulted in a significant attenuation of the transformation of activated microglia into an amoeboid shape.

S1P_3_ in the ischemic brain may also link into astrogliosis, a core pathogenesis associated with inflammatory responses in cerebral ischemia [23]. In fact, S1P microinjection into the brain has been reported to cause astrogliosis [6, 51]. Recently, S1P_3_ was identified as the receptor subtype to regulate astrogliosis, in which a pharmacological antagonism or genetic deletion of S1P_3_ reduced S1P-triggered inflammatory responses in astrocytes [24]. These previous findings indicate that S1P_3_-triggered astrogliosis may occur in an ischemic brain. Indeed, we demonstrated that inhibiting S1P_3_ after tMCAO challenge resulted in a significant attenuation of astrogliosis.

The phenotypical shift of activated microglia has also been extensively considered to understand the pathogenesis of cerebral ischemia [25]. Activated microglia in the ischemic brain become polarized to different phenotypes: classically activated M1- or alternatively activated M2-polarized microglia [25]. M1 microglia are considered as toxic and proinflammatory cells in diverse CNS disorders including cerebral ischemia [52], and the prevention of toxic transformation towards M1 phenotypes has been considered as a possible therapeutic strategy for cerebral ischemia [53, 54]. In contrast, M2-polarized microglia are involved in the repair and resolution phase of ischemic recovery, leading to the neuroprotection [53]. In this study, the suppression of S1P_3_ activity in the ischemic brain attenuated M1 polarization, as evidenced by the attenuated gene expression of relative markers following tMCAO. However, S1P_3_ suppression did not alter the expression levels of M2 polarization-relevant markers following tMCAO. These data demonstrate that S1P_3_ in the ischemic brain is selectively associated with the M1 polarization. This unique role of S1P_3_ in M1 polarization was obvious in activated microglia, which was confirmed by determining the expression levels of microglial NF-κB, a characteristic marker for M1 polarization [54]. Inhibiting S1P_3_ significantly reduced the number of Iba1/p65 NF-κB double-immunopositive cells. These in vivo findings of the link between S1P_3_ and M1 microglial polarization were further affirmed in LPS-stimulated mouse primary microglia, in which inhibiting S1P_3_ by both genetic and pharmacological tools ensured the attenuation of proinflammatory cytokines. Therefore, S1P_3_ may mediate brain injury following tMCAO by altering the microglial polarization states to M1, further suggesting that S1P_3_ is a novel and selective player in regulating M1 microglial polarization.

The underlying signaling mechanisms for the pathogenic roles of S1P_3_ in cerebral ischemia were linked to PI3K/Akt and MAPK pathways, including ERK1/2 and p38 MAPK. Inhibiting S1P_3_ following tMCAO increased the Akt phosphorylation in the ischemic brain, whereas it attenuated the phosphorylation of ERK1/2 and p38 MAPK. These signaling molecules are, in particular, considered to regulate the phenotype shift between M1 and M2 polarization. Akt activation in microglia is a signaling molecule that drives activated microglia towards M2 polarization. Additionally, the activation of PI3K/Akt signaling is critical for restricting inflammatory activation of microglia/macrophages and negatively regulates NF-κB signaling, whereas its inhibition drives activated microglia/macrophages towards their M1 polarization [31, 55]. In this context, Akt activation is crucial for cell phenotype shift by inhibiting M1 and activating M2 polarization. In this study, the suppression of S1P_3_ activity in ischemic brain increased Akt phosphorylation without altering the expression markers of M2 polarization, indicating that the increased Akt phosphorylation by S1P_3_ inhibition may be linked to the restriction of M1 polarization rather than to the enhancing of M2 polarization. Persistent activation of ERK1/2 signaling has been reported to trigger NF-κB transcriptional activity [28, 29] similar to the activation of p38 [30], both of which eventually lead to the secretion of proinflammatory mediators that are associated with the M1 polarization of activated microglia [56–58]. This further ensured that S1P_3_ activation is closely associated with the M1 polarization of activated microglia in the ischemic brain because the suppression of S1P_3_ activity attenuated ERK1/2 and p38 MAPK phosphorylation in the ischemic brain.

## Conclusions

This study identified S1P_3_ as a novel pathogenic factor in cerebral ischemia and provided underlying mechanisms, particularly in view of microglial activation. The medically relevant roles of the S1P receptor subtypes in cerebral ischemia have emerged through translational studies. Now, at least three subtypes have been identified to mediate brain injury in cerebral ischemia, including S1P_1_ [13], S1P_2_ [14], and S1P_3_ (the current study). Even though S1P_3_ may be limited as a therapeutic target because of its negative effects on the heart, it would be a good therapeutic strategy for cerebral ischemia if S1P_3_-specific antagonist can act inside the CNS. In addition to the identification of novel roles of S1P_3_, our findings also implicate that the neuroprotective effects exerted by FTY720 in cerebral ischemia in previous studies occur additionally via suppressing S1P_3_ activity [17], similar to the case of S1P_1_ [13, 59, 60].

## Additional file

Additional file 1: **Figure S1.** CAY10444 (CAY) administration attenuates tMCAO-induced neurodegeneration in post-ischemic brain. **Figure S2.** CAY10444 (CAY) administration does not alter tMCAO-induced microglial M2 polarization in post-ischemic brain. **Figure S3.** CAY10444 (CAY) administration attenuates tMCAO-induced astrocytes activation in post-ischemic brain. **Table S1.** Primer sets used for qRT-PCR analysis. (DOCX 1425 kb)

## Abbreviations

ABC: Avidin and biotinylated horse-radish peroxidase macromolecular complex
BrdU: Bromodeoxyuridine
CNS: Central nervous system
DAB: 3, 3′-diaminobenzidine tetrahydrochloride
ECL: Enhanced chemiluminescence
FBS: Fetal bovine serum
FJB: Fluoro Jade B
GFAP: Glial fibrillary acidic protein
LPS: Lipopolysaccharides
mNSS: Modified neurological severity score
NPER: Neuronal protein extraction reagent
PBS: Phosphate-buffered saline
S1P: Sphingosine 1-phosphate
tMCAO: Transient middle cerebral artery occlusion
TTC: 2,3,5-Triphenyltetrazolium chloride
