# Supplementary material for: Sphingosine 1-phosphate receptor subtype 3 (S1P3) contributes to brain injury after transient focal cerebral ischemia via modulating microglial activation and their M1 polarization
Source: J Neuroinflammation. 2018 Oct 10;15:284. doi: 10.1186/s12974-018-1323-1 (PMC6180378; doi:10.1186/s12974-018-1323-1)
Supplement: Supplementary file 1 — Figure S1. CAY10444 (CAY) administration attenuates tMCAO-induced neurodegeneration in post-ischemic brain. Figure S2. CAY10444 (CAY) administration does not alter tMCAO-induced microglial M2 polarization in post-ischemic brain. Figure S3. CAY10444 (CAY) administration attenuates tMCAO-induced astrocytes activation in post-ischemic brain. Table S1. Primer sets used for qRT-PCR analysis. (DOCX 1425 kb) [file 12974_2018_1323_MOESM1_ESM.docx]

**Sphingosine 1-phosphate receptor subtype 3 (S1P_3_) contributes to brain injury after transient focal cerebral ischemia *via* modulating microglial activation and their M1 polarization**

Bhakta Prasad Gaire^1^, Mi-Ryoung Song^2,#^, Ji Woong Choi^1,#^

^1^College of Pharmacy and Gachon Institute of Pharmaceutical Sciences, Gachon University, Incheon 406-799, Republic of Korea; ^2^School of Life Sciences, Gwangju Institute of Science and Technology, Gwangju 500-712, Republic of Korea

Running title: Pathogenic roles of S1P_3_ in cerebral ischemia.

^#^Co-corresponding author:

Ji Woong Choi, Ph.D.

Laboratory of Neuropharmacology, College of Pharmacy, Gachon University, Yeonsu-gu, Incheon 406-799, Republic of Korea; Tel: +82-32-820-4955; Fax: +82-32-820-4829; E-mail: [pharmchoi@gachon.ac.kr](mailto:pharmchoi@gachon.ac.kr)

Mi-Ryoung Song, Ph.D.

School of Life Sciences, Gwangju Institute of Science and Technology, Republic of Korea Buk-gu, Gwangju 500-712, Republic of Korea; Tel: +82-62-715-2508; Fax: +82-62-715-2484; E-mail: [msong@gist.ac.kr](mailto:msong@gist.ac.kr)

**Supporting Information:**

**
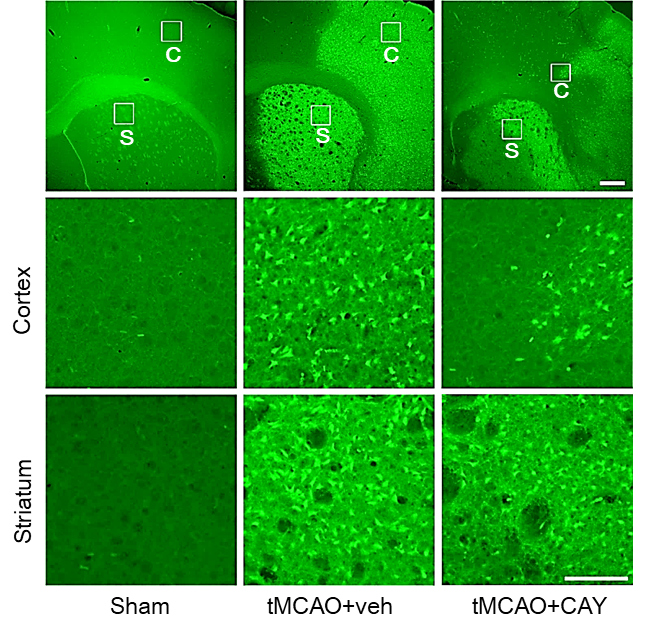
**

**Fig. S1 CAY10444 (CAY) administration attenuates tMCAO-induced neurodegeneration in post-ischemic brain.** Mice were challenged with tMCAO, and CAY (0.5 mg/kg) was administered intraperitoneally immediately after reperfusion. The effect of CAY on neuronal cell death was assessed 24 h after reperfusion by Fluoro Jade B (FJB) staining. Representative images of FJB-stained ipsilateral brain sections. Scale bars, 200 μm (top panels) and 50 μm (middle and bottom panels). n = 5 mice per group.

**
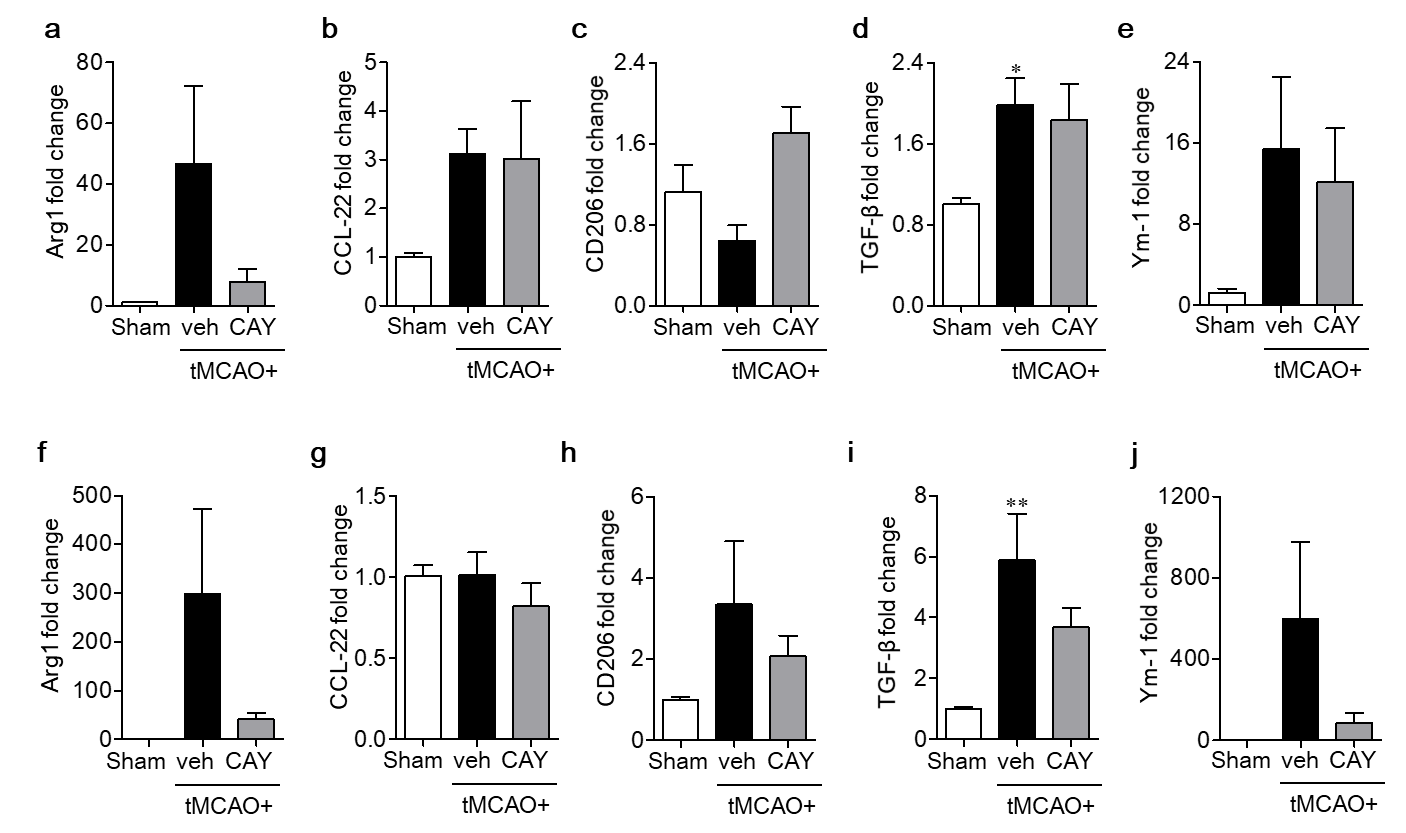
**

**Fig. S2 CAY10444 (CAY) administration does not alter tMCAO-induced microglial M2 polarization in post-ischemic brain.** Mice were challenged with tMCAO, and CAY (0.5 mg/kg) was administered intraperitoneally immediately after reperfusion. The effect of CAY on mRNA expression of M2-polarized microglial markers in 1 and 3 days post-ischemic brain was determined by qRT-PCR analysis. (a-d) Expression of surface markers of M2-polarized microglia in 1 day post-ischemic brain, and (e-h) expression of surface markers of M2-polarized microglia in 3 days post-ischemic brain. n = 5 mice per group. **p*<0.05 and ***p*<0.01, versus sham group.

**
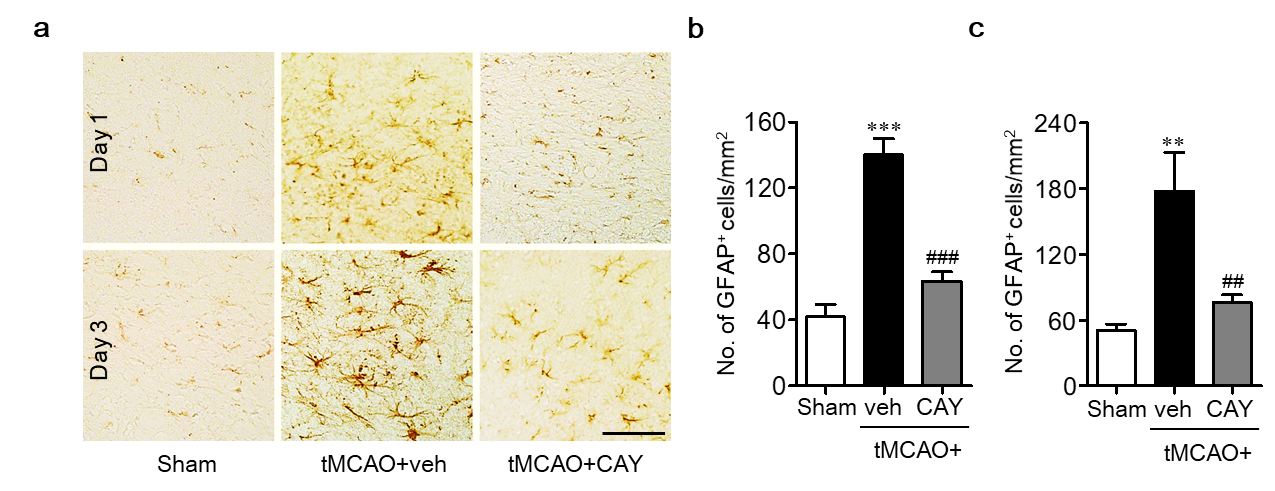
**

**Fig. S3 CAY10444 (CAY) administration attenuates tMCAO-induced astrocytes activation in post-ischemic brain.** Mice were challenged with tMCAO, and CAY (0.5 mg/kg) was administered intraperitoneally immediately after reperfusion. The effect of CAY on astrocytes activation was determined by GFAP immunohistochemistry in 1 and 3 days post-ischemic brain. (a) Representative images of GFAP-immunopositive cells in the corpus callosum. Scale bar, 50 μm. Quantification of the number of GFAP-immunopositive cells in 1 day (b) and 3 days (c) post-ischemic brain. n = 4~5 mice per group. ***p*<0.01 and ****p*<0.001 versus sham. ^##^*p*<0.01 and ^###^*p*<0.001 versus vehicle-administered tMCAO group (tMCAO+veh).

**Table S1. Primer sets used for qRT-PCR analysis**

| Target | Direction | Sequence | Gene Accession # |
| --- | --- | --- | --- |
| β-actin | Forward | 5ˈ-AGCCTTCCTTCTTGGGTATG-3ˈ | NM_007393 |
|  | Reverse | 5ˈ-CTTCTGCATCCTGTCAGCAA-3ˈ |  |
| TNF-α | Forward | 5ˈ-CATCTTCTCAAAATTCGAGTGACAA-3ˈ | NM_013693 |
|  | Reverse | 5ˈ-TGGGAGTAGACAAGGTACAACCC-3ˈ |  |
| IL-1β | Forward | 5ˈ-CAACCAACAAGTGATATTCTCCATG-3ˈ | NM_008361 |
|  | Reverse | 5ˈ-GATCCACACTCTCCAGCTGCA-3ˈ |  |
| IL-6 | Forward | 5ˈ-GAGGATACCACTCCCAACAGACC-3ˈ | NM_031168 |
|  | Reverse | 5ˈ-AAGTGCATCATCGTTGTTCATACA-3ˈ |  |
| S1P_3_ | Forward | 5ˈ-TTGCAGAACGAGAGCCTATT-3ˈ | NM_010101 |
|  | Reverse | 5ˈ-TTCCCGGAGAGTGTCATTTC-3ˈ |  |
| CD11b | Forward | 5ˈ-CCCCAATTACGTAGCGAATG-3ˈ | NM_001082960 |
|  | Reverse | 5ˈ-TGCTGCGAAGATCCTAGTTG-3ˈ |  |
| CD16 | Forward | 5ˈ-TATGGCACCTTAGCGTGATG-3ˈ | NM_010188 |
|  | Reverse | 5ˈ-CGACCCTGTAGATCTGGGAG-3ˈ |  |
| CD32 | Forward | 5ˈ-CTCGAGTTTGACCACAGCCT-3ˈ | NM_001077189 |
|  | Reverse | 5ˈ-TGTTCTCACGGACTTTGTGC-3ˈ |  |
| CD86 | Forward | 5ˈ-TCTCCACGGAAACAGCATCT-3ˈ | NM_019388 |
|  | Reverse | 5ˈ-CTTACGGAAGCACCCATGAT-3ˈ |  |
| Arg1 | Forward | 5ˈ-TTTTTCCAGCAGACCAGCTT-5ˈ | NM_007482 |
|  | Reverse | 5ˈ-AGAGATTATCGGAGCGCCTT-3ˈ |  |
| CCL-22 | Forward | 5ˈ-TGGAGTAGCTTCTTCACCCA-3ˈ | NM_009137 |
|  | Reverse | 5ˈ-TCTGGACCTCAAAATCCTGC-3ˈ |  |
| CD206 | Forward | 5ˈ-GTGGATTGTCTTGTGGAGCA-3ˈ | NM_008625 |
|  | Reverse | 5ˈ-TTGTGGTGAGCTGAAAGGTG-3ˈ |  |
| TGF-β1 | Forward | 5ˈ-CAACCCAGGTCCTTCCTAAA-3ˈ | NM_011577 |
|  | Reverse | 5ˈ-GGAGAGCCCTGGATACCAAC-3ˈ |  |
| Ym1 | Forward | 5ˈ-TTTCTCCAGTGTAGCCATCCTT-3ˈ | NM_009892 |
|  | Reverse | 5ˈ-AGGAGCAGGAATCATTGACG-3ˈ |  |
